# Co‐expression network analysis of diverse wheat landraces reveals markers of early thermotolerance and a candidate master regulator of thermotolerance genes

**DOI:** 10.1111/tpj.16248

**Published:** 2023-05-20

**Authors:** Liam J. Barratt, Zhesi He, Alison Fellgett, Lihong Wang, Simon McQueen Mason, Ian Bancroft, Andrea L. Harper

**Affiliations:** ^1^ Department of Biology, Centre for Novel Agricultural Products (CNAP) University of York Wentworth Way YO10 5DD UK

**Keywords:** network analysis, *Triticum aestivum*, landrace, hub gene, heat stress, thermotolerance

## Abstract

*Triticum aestivum* L. (bread wheat) is a crop relied upon by billions of people around the world, as a major source of both income and calories. Rising global temperatures, however, pose a genuine threat to the livelihood of these people, as wheat growth and yields are extremely vulnerable to damage by heat stress. Here we present the YoGI wheat landrace panel, comprising 342 accessions that show remarkable phenotypic and genetic diversity thanks to their adaptation to different climates. We quantified the abundance of 110 790 transcripts from the panel and used these data to conduct weighted co‐expression network analysis and to identify hub genes in modules associated with abiotic stress tolerance. We found that the expression of three hub genes, all heat‐shock proteins (HSPs), were significantly correlated with early thermotolerance in a validation panel of landraces. These hub genes belong to the same module, with one (*TraesCS4D01G207500.1*) being a candidate master‐regulator potentially controlling the expression of the other two hub genes, as well as a suite of other HSPs and heat‐stress transcription factors (HSFs). In this work, therefore, we identify three validated hub genes, the expression of which can serve as markers of thermotolerance during early development, and suggest that *TraesCS4D01G207500.1* is a potential master regulator of HSP and HSF expression – presenting the YoGI landrace panel as an invaluable tool for breeders wishing to determine and introduce novel alleles into modern varieties, for the production of climate‐resilient crops.

## Significance Statement

Global wheat yields and crop growth are vulnerable to damage by heat stress, a threat that is likely to become more common globally in the coming years through climate change. Conducting weighted co‐expression network analysis on 337 diverse landrace accessions, we identified and validated *TraesCS4D01G207500.1* as a hub gene that can be used as a marker of early thermotolerance, seemingly via the regulation of the expression of a suite of HSP and HSF genes.

## INTRODUCTION

*Triticum aestivum* L. (bread wheat) is one of the most important crops worldwide, accounting for 20% of all calories consumed annually (Pfeifer et al., 2014; Food and Agriculture Organisation of the United Nations et al., 2018). However, with the global population expected to increase to 9 billion by 2050, at least a 50% increase in crop yields must be achieved within the next three decades (Godfray et al., 2010; Ray et al., 2013; Tilman et al., 2011). A major obstacle in the way of this increase is the changing climate, as rising global temperatures has meant that seasonal periods of extreme heat stress are becoming more common and water supplies are becoming heavily depleted (Hansen et al., 2006). This is particularly worrying for those dependent on wheat yields for their nutrition and/or income, as heat stress is especially damaging to wheat growth. As a cool season crop, wheat has an optimal growth temperature of around 20°C and shows a 3–6% reduction in yield for every degree above this optimum (Chowdhury & Wardlaw, 1978; Kobza & Edwards, 1987; Nagai & Makino, 2009; Ray et al., 2013; Tian et al., 2018; Wardlaw et al., 1989; Zhao et al., 2017). With the Intergovernmental Panel on Climate Change (IPCC) predicting that global mean surface temperatures towards the end of the century will be between 0.3°C and 4.8°C higher than they were a century prior (Collins et al., 2013), and with some models predicting that the earth's average global temperature may rise by 2°C–5°C by the year 2060 (De Costa, 2011; Murphy et al., 2004; Wigley & Raper, 2001), wheat crops are likely to be under considerable risk of damage through heat stress in the coming years. Therefore, the cultivation of thermotolerant wheat varieties is of paramount importance if crops are to be protected against an increasingly hostile climate.

High‐yielding elite varieties, produced during and after the Green Revolution, are used both as commercial food crops and in breeding programmes around the world, but show reduced genetic diversity and an absence of alleles encoding novel traits, such as abiotic stress tolerance (Fu, 2015; Keneni et al., 2012; Pingali, 2012; Tanksley & McCouch, 1997). This loss of genetic diversity comes as a result of breeding programmes, which act as significant genetic bottlenecks, reducing genetic diversity in search of favourable traits, such as high yields (Fu, 2015; Keneni et al., 2012; Tanksley & McCouch, 1997). Wheat landraces, however, are locally grown varieties that have adapted, through a combination of natural selection and small‐scale selection by farmers, to grow successfully in their local climate (Zeven, 1998). These landraces originate from a variety of different countries and, subsequently, climates, so exhibit great variation in their degree of abiotic stress tolerance. This phenotypic diversity is borne out of the remarkable genetic diversity shown by landrace varieties, as a result of their wide geographical distribution into distinct populations, and the absence of major genetic bottlenecks, such as intense breeding programmes, in their recent ancestry. The combination of phenotypic and genetic diversity means wheat landraces are a valuable resource for breeders in the production of varieties more resilient to the challenges posed by a changing climate.

Traditionally, conventional breeding approaches have been used in an attempt to improve the abiotic stress tolerance of wheat varieties (Manès et al., 2012; Pfeifer et al., 2005; Schmidt, 1983). The improvements made using such methods, however, are often marginal and slow, largely because of their untargeted nature (Manès et al., 2012; Pfeifer et al., 2005). More recently, powerful statistical genetics approaches such as genome‐wide association studies (GWASs) have been employed to identify genetic markers (single‐nucleotide polymorphisms, SNPs) associated with traits of interest, enabling the identification of candidate genes and the selection of beneficial germplasm at the seedling stage, using marker‐assisted breeding (MAB) (Abou‐Elwafa & Shehzad, 2021; Li et al., 2019; Mathew et al., 2019; Maulana et al., 2020). Although such approaches have proven successful, the sequence markers identified using GWASs are often not causal, instead highlighting regions of the genome in high‐linkage disequilibrium with the causal gene. This approach, therefore, can frequently provide more questions than answers in terms of understanding the biological mechanism linking marker and trait.

The link between marker and trait is much clearer for gene expression markers, however, with differences in gene expression invariably affecting traits directly. The identification of such markers has become increasingly common over the past decade, through improvements in RNA‐sequencing (RNA‐seq) technology allowing comprehensive studies into the effect of abiotic stress on the expression of the entire wheat transcriptome (Chu et al., 2021; Iquebal et al., 2019; Lv et al., 2018; Ma et al., 2017).

Although such studies can identify a wealth of differentially expressed genes, selecting and screening the most promising candidates for abiotic stress tolerance improvement can be laborious and time‐consuming. To circumvent this, and quickly identify the most promising candidate genes, network approaches are increasingly being used in crop species. The creation of these networks has allowed the relationships between key factors in biological processes to be elucidated and master regulators within systems to be identified (Pavlopoulos et al., 2011). Weighted gene co‐expression network analysis (WGCNA) (Langfelder & Horvath, 2008, 2012) is the most commonly used network approach, which establishes a correlation gene network to group genes with similar expression patterns, across all samples, into modules. These modules often house genes involved in the same, or similar, biochemical or physiological processes, and, subsequently, the identification of the most central genes within a module provides an insight into which genes may be the master regulators of the other genes in the module and, in turn, these processes. Manipulating the activity or expression of these central genes (or ‘hub genes’) is likely to have a dramatic effect on the phenotype, as the expression of all genes under the influence of the regulator will also be affected by the modification. Despite the power of this method, however, network approaches have been used sparingly to study thermotolerance in wheat (Girousse et al., 2018; Mishra et al., 2021).

In the present work, we sequenced transcriptomes of a panel of bread wheat landraces (hereafter referred to as the YoGI panel), selected from multiple germplasm collections to represent a large selection of wheat‐growing regions and environmental conditions. Thermotolerance hub genes housed within stress‐associated modules, from a co‐expression network created using expression data from the YoGI panel, were then identified and validated as markers for early thermotolerance. In addition, transcriptome display tile plots (TDTPs) were interrogated to discern whether naturally occurring rearrangements and homoeologous exchanges have occurred in any of the landrace accessions. Homoeologous exchanges, where recombination occurs between homoeologous (chromosomes from different subgenomes) rather than homologous chromosomes, can result in segmental deletions or duplications in one of the subgenomes, which could potentially affect thermotolerance if they affect hub gene locations.

## RESULTS

### Co‐expression network construction and network conformation statistics

RNA‐seq data were mapped to the IWGSC RefSeq transcriptome v1.0 coding sequence (CDS) models; mapping statistics are provided in Table S1. Normalized transcript abundance (reads per kilobase of transcript per million mapped reads, RPKM) for each of the landraces and diploid relatives is provided in Table S2. The co‐expression network contained 324 modules, housing 94 057 genes. The mean module size was 291 genes, whereas the median module size was 56 genes. Three modules shared the smallest number of genes (30), whereas the largest module contained 6923 genes.

### Identifying stress‐associated modules

Modules enriched in Gene Ontology (GO) terms related to responses to heat, abiotic stress or abiotic stimuli are likely to contain genes that determine a plant's degree of stress tolerance. Thirteen of the 324 modules were found to be significantly enriched in such GO terms (Table 1), with the blue and lavenderblush1 modules being enriched in GO terms directly related to thermotolerance (‘response to heat’, with false discovery rates, FDRs, of 0.00011 and 1.5e‐09, respectively). Three of the 13 modules were enriched in the GO term ‘response to water’ – these modules were studied in the present work, as heat and drought stress can often occur simultaneously during periods of high temperature, so tolerance to drought stress may also bring thermotolerance.

**Table 1:** Thirteen modules were significantly enriched in stress‐related Gene Ontology (GO) terms, according to GO enrichment analysis using the singular enrichment analysis tool in agrigo (Du et al., 2010; Tian et al., 2017). The names of these modules and the enriched stress‐related GO terms are listed. The FDR‐adjusted Fisher's test *P*‐values associated with each GO term are given in brackets

Module	Enriched GO term
Blue	Response to heat (0.0001)
Darkgrey	Response to stress (0.009)
Darkmagenta	Response to oxidative stress (0.0002)
Darkorange	Response to water (1.4e‐05)
Lavenderblush1	Response to heat (1.5e‐09)
Lightyellow	Response to water (2.8e‐19)
Mediumpurple	Response to stress (0.004)
Navajowhite3	Response to stimulus (0.031)
Purple	Response to stress (9.4e‐06)
Skyblue	Response to water (5.9e‐06)
Skyblue2	Response to stress (0.02)
Steelblue	Response to stress (0.0008)
Thistle	Response to stress (0.022)

### Hub gene identification

Within the 13 stress‐associated modules, several hub genes showed sequence similarity to stress tolerance genes in wheat or other species (Table 2). These hub genes were particularly promising as they have been directly implicated in thermotolerance, acting as heat‐shock proteins (HSPs), involved in governing normal growth and development, orchestrating stress hormone signalling or acting as transcription factors which are likely to have far‐reaching effects on the expression of other genes.

**Table 2:** Hub genes identified in stress‐associated modules

Gene	BLAST Hit	Function	Reference
***TraesCS5A01G105900.1***	***Ttriticum aestivum UBP12 (100%)***	**Jasmonic acid and abscisic acid signalling**	Jeong et al. (2017 and Liu et al. (2022)
*TraesCS7A01G050400.1*	*TaClpB1‐like (99.95%)*	Long‐term acquired thermotolerance	Mishra and Grover (2016)
*TraesCS6A01G158100.1*	*TaMADS‐box transcription factor 29‐like (100%)*	MADS‐box transcription factor	Castelán‐Muñoz et al. (2019)
*TraesCS5A01G369900.1*	*TaCS66‐like (100%)*	Cold‐shock protein	Park et al. (2009)
***TraesCS5D01G125500.1***	***Aegilops tauschii DnaJ A7A*, *chloroplastic‐like (100%)***	**Chloroplast development**	Zhu et al. (2015)
*TraesCS1A01G314200.1*	*TaABI4‐like (100%)*	Stress‐hormone signalling	Chandrasekaran et al. (2020)
*TraesCS3B01G270800.1*	*TaLPP2 (100%)*	ABA signalling	Paradis et al. (2011)
*TraesCS3B01G285100.1*	*TaLEA14 (97.79%)*	Protection against abiotic stress	Hong‐Bo et al. (2005 and Jia et al. (2014)
*TraesCS2A01G447400.1*	*TaMYB34‐like (100%)*	Stress‐responsive transcription factor	Wang et al. (2021)
***TraesCS2B01G205600.1***	***TdPELPK1‐like (97.33%)***	**Positive influence on growth**	Rashid and Deyholos (2011)
***TraesCS4D01G207500.1***	***AtHSP70‐1 (78%)***	**Negative influence on heat‐stress tolerance**	Tiwari et al. (2020)
***TraesCS7B01G149200.1***	***TaHsp90.2‐B1 (100%)***	**Long‐term acquired thermotolerance**	Kumar and Rai (2014)
***TraesCS7D01G241100.1***	***TaHsp90.2‐D1 (100%)***	**Long‐term acquired thermotolerance**	Kumar and Rai (2014)

These hub genes were deemed to be particularly promising, based on the known function of the hub gene itself, its orthologue in other species or the general functions of its protein family. The hub genes in bold were studied further as part of validation experiments. BLAST percentage identity scores are given in brackets after each BLAST hit.

**Figure 1:**
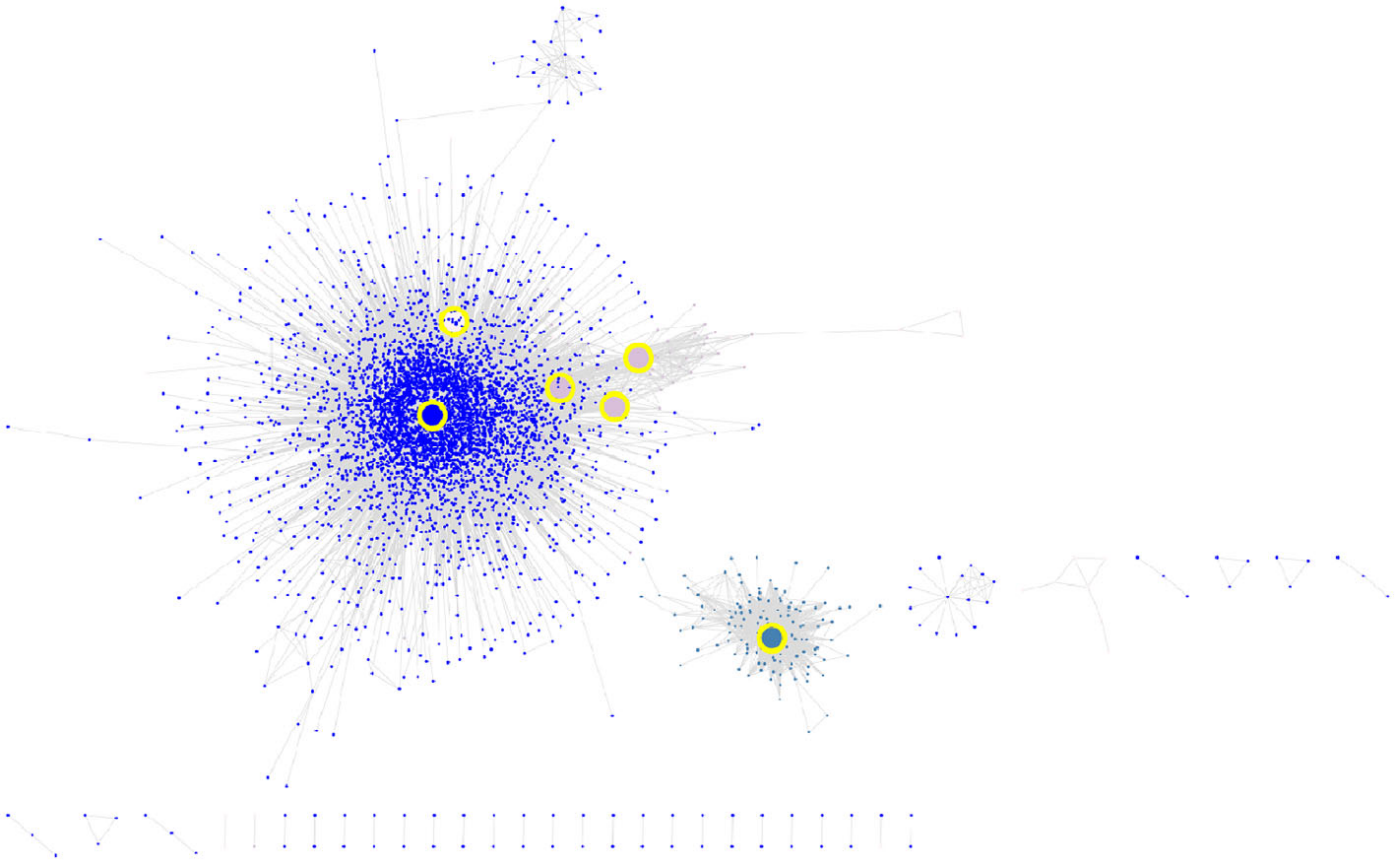
Subnetwork of the modules containing the six stress‐associated hub genes used to predict thermotolerance. Stress‐associated modules housing seven genes used to predict thermotolerance were exported together using the ‘exportNetworkToCytoscape()’ function, with a threshold of 0.1, before the subnetwork was visualized in cytoscape. Node colour corresponds to the module membership of each gene, and the six hub genes are enlarged and highlighted with a yellow border. Each node represents a gene in the subnetwork, whereas each line between the nodes represents a connection between the genes.

Six of the 13 promising hub genes had a mean RPKM > 1 across the panel, so these were the hub genes tested as markers of thermotolerance (Figures 1 and S1). Despite being unable to be tested as markers in the present work, the remaining seven hub genes represent valuable targets for wheat breeders in the development of thermotolerant varieties, where these genes are expressed at a higher level, as their centrality within stress‐associated modules in the network suggests that the manipulation of their expression may have large effects on global gene expression, and potentially on thermotolerance.

### Transcriptome display tile plots

**Figure 2:**
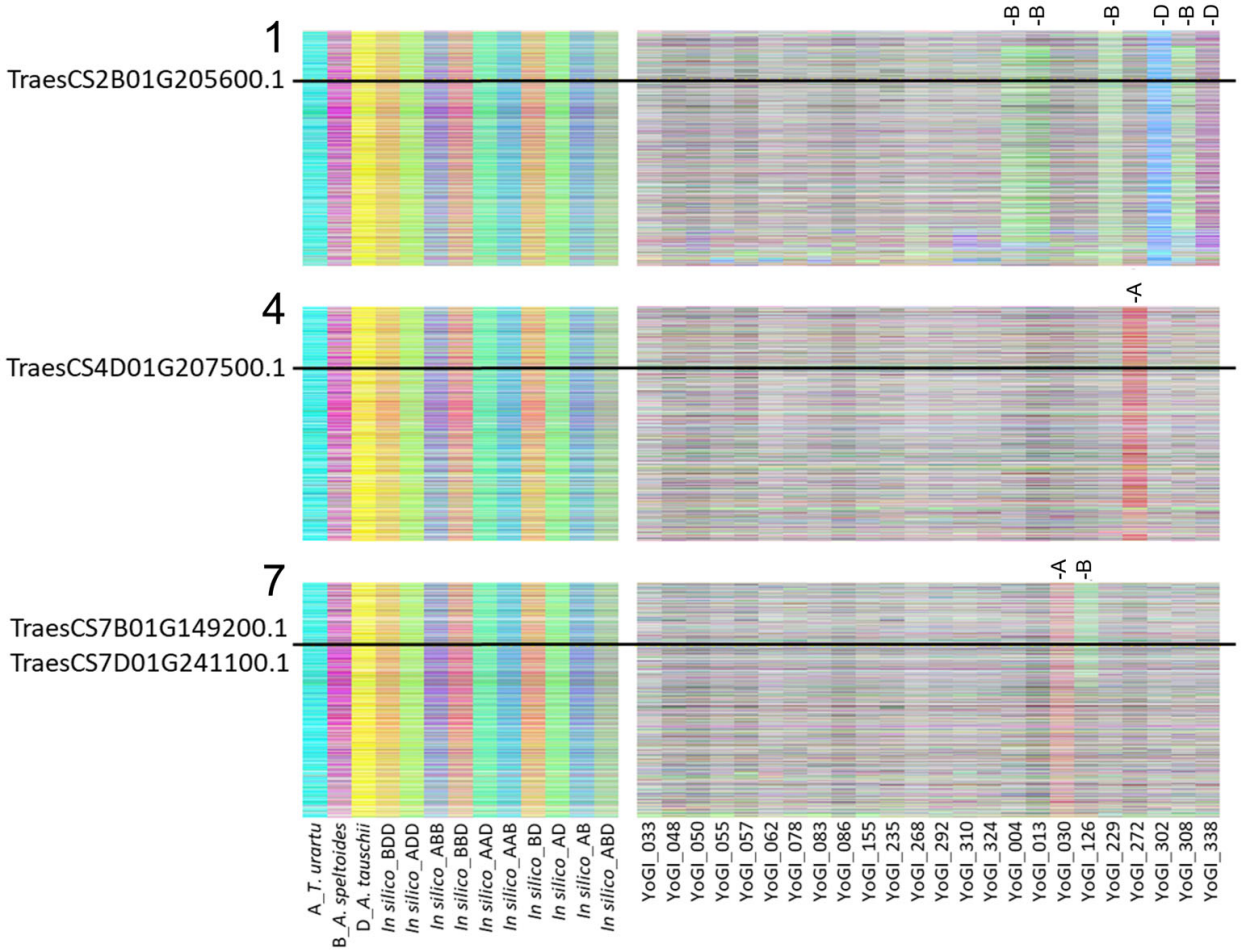
Transcriptome display tile plot for the YoGI wheat landrace panel. Tile plots illustrate relative transcript contributions for the A, B and D copies of 15 527 triplets of homoeologous genes on linkage group 2. Represented are 342 bread wheat accessions, diploid ancestors *Triticum urartu* (AA), *Aegilops speltoides* (BB) and *Aegilops tauschii* (DD), tetraploid ancestor *Triticum dicoccoides* (AABB) and *in silico* tetra‐ and hexaploid combinations. The A genome is represented in cyan, the B genome is represented in magenta and the D genome is represented in yellow. The homoeologous genes are arranged in A genome order.

To assess whether naturally occurring rearrangements or homoeologous exchanges (HEs) in the wheat genome may have the potential to affect the thermotolerance of different landraces, we compared candidate hub gene locations with these events using TDTPs, which enable the visual comparison of expression for the three wheat genomes, to identify regions where HEs or other rearrangements have occurred. As in previous studies (Harper et al., 2016; He et al., 2017), transcript abundance of homoeologous triads (21 073 in total) was visually examined after being assigned to colour space, where the A genome is represented in cyan, the B genome is represented in magenta and the D genome is represented in yellow (Figure 2).

As expected for a panel of diverse lines, a large number of structural rearrangements were detected in the landraces, varying greatly in size. Of the six hub genes, four were found in rearranged regions in the landrace panel. In YoGI_272, it appears that a rearrangement has occurred in the collinear region encompassing *TraesCS4D01G207500.1*, with only the B and D genome copies of this region being present. *TraesCS7B01G149200.1* and *TraesCS7D01G241100.1* are homoeologues, and this collinear region also appears to be affected by rearrangements in YoGI_30 and YoGI_126 accessions. YoGI_030 appears to be missing the entire 7A chromosome, and a segmental rearrangement also appears to have affected YoGI_126, removing the B genome homoeologue of this gene. Finally, large rearrangements were found in the collinear region surrounding *TraesCS2B01G205600.1* in six of the landraces. YoGI_004, 013, 229 and 308 appear to possess only the A and D genomes in this region, YoGI_302 appears to have only A genome expression in this region and YoGI_338 has only B genome expression in this region. Where these rearrangements have occurred, in some cases the TDTPs also indicate a potential increased contribution from one of the remaining genomes.

We found that although these lines were not outliers, in all cases the total triad transcript abundance (RPKM) for lines with these rearrangements is lower than the average for lines without them, consistent with reduced hub gene dosage in accessions affected by these rearrangements (Table 3). Despite the potential for RNA‐seq reads to align with all subgenomes when there is high sequence similarity among homoeologues, in general the RPKM values are also lower for the homoeologue that the TDTP indicates is missing. As the hub genes are positively correlated with heat tolerance, we would predict average or poorer levels of heat tolerance in all of these accessions. In addition, as relatively few accessions have rearrangements in these regions, we elected to choose accessions to validate the predictive capability of the gene network hubs based on hub RPKM values only.

**Table 3:** Reads per kilobase of transcript per million mapped reads (RPKM) values for hub‐gene triads with chromosome rearrangements detected

	*TraesCS4A01G097900.1*	*TraesCS4B01G206700.1*	***TraesCS4D01G207500.1***	Total
Mean RPKM (no rearrangement; *n* = 341)	51.148	74.773	131.740	257.661
RPKM YoGI_272 (−A)	13.879	60.184	110.299	184.361
	*TraesCS2A01G178600.1*	***TraesCS2B01G205600.1***	***TraesCS2D01G187300.1***	Total
Mean RPKM (no rearrangement; *n* = 340)	29.359	38.707	28.629	96.695
RPKM YoGI_030 (−A)	15.327	34.286	27.003	76.616
RPKM YoGI_126 (−B)	36.317	22.785	33.141	92.244
	*TraesCS2A01G178600.1*	***TraesCS2B01G205600.1***	*TraesCS2D01G187300.1*	Total
Mean RPKM (no rearrangement; *n* = 336)	0.003	4.053	5.242	9.298
RPKM YoGI_004 (−B)	0.000	0.000	3.438	3.438
RPKM YoGI_013 (−B)	0.000	0.000	3.779	3.779
RPKM YoGI_229 (−B)	0.000	0.327	1.014	1.341
RPKM YoGI_302 (−D)	0.000	2.721	0.000	2.721
RPKM YoGI_308 (−B)	0.000	0.000	3.635	3.635
RPKM YoGI_338 (−D)	0.000	1.525	0.660	2.186

Hub genes detected using WGCNA are indicated in bold, and missing genome contributions predicted by the TDTP are indicated next to accession numbers.

### Hub gene validation

A total of 15 spring habit landrace accessions were selected from the panel for use in a thermotolerance validation assay, based on the variation that they showed in hub gene RPKM (Table S4). These accessions showed an average normalized loss in mean dry biomass of 0.364 when exposed to heat stress during early development, compared with their counterparts under control conditions. The most tolerant accession showed a normalized loss in mean dry biomass of 0.135 (YoGI_155), whereas the most susceptible accession (YoGI_268) showed a score of 0.513.

**Figure 3:**
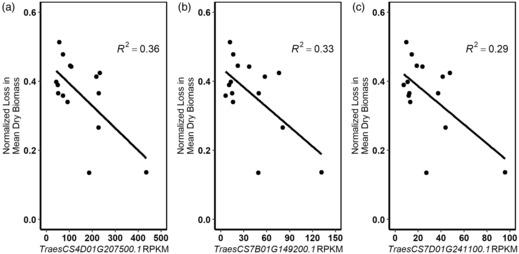
The expression levels of three heat‐shock protein (HSP) hub genes were significantly associated with early thermotolerance. The associations between hub gene reads per kilobase of transcript per million mapped reads (RPKM) values and normalized loss in mean dry biomass shown by a small panel of landrace accessions were analysed by linear regression. Significant associations were seen for three hub genes: (a) *TraesCS4D01G207500.1* (*R* ^2^ = 0.36, *P* = 0.011), (b) *TraesCS7B01G149200.1* (*R* ^2^ = 0.33, *P* = 0.015) and (c) *TraesCS7D01G241100.1* (*R* ^2^ = 0.29, *P* = 0.021).

To determine whether hub gene RPKM could be used as a marker of early thermotolerance, regression analyses were conducted, comparing the RPKM of each hub gene, shown by each accession, with the normalized loss in mean dry biomass shown by each accession. We hypothesized that if the expression of a hub gene under control conditions could be used as a marker for early thermotolerance, a significant relationship between hub gene RPKM and normalized dry biomass loss would be observed. This was the case for three of the six hub genes (Figure 3), all of which were members of the same module: *TraesCS4D01G207500.1* (*R* ^2^ = 0.36, *P* = 0.011), *TraesCS7B01G149200.1* (*R* ^2^ = 0.33, *P* = 0.015) and *TraesCS7D01G241100.1* (*R* ^2^ = 0.29, *P* = 0.021). No significant relationship was observed between hub gene RPKM and normalized dry biomass loss for the remaining three hub genes, however (*P* > 0.05, Figure S1). A significant relationship was observed when comparing the expression of the most central gene in the module, *TraesCS4D01G207500.1*, with *TraesCS7B01G149200.1* (*R* ^2^ = 0.94, *P* = 1.11e‐09) and *TraesCS7D01G241100.1* (*R* ^2^ = 0.96, *P* = 9.18e‐11), suggesting that *TraesCS4D01G207500.1* is likely to regulate the expression of the two homoeologous hubs.

## DISCUSSION

The transition of agriculture towards genetic uniformity may have resulted in higher, more reliable yields under optimal conditions, but as the climate becomes increasingly hostile for cereal crop cultivation, the need to recapture some of the genetic diversity lost during this transition is essential to prepare future varieties for growth under such challenging conditions. Landrace varieties are a valuable source of both phenotypic and genetic diversity, through their adaptation to climates around the world and the lack of major genetic bottlenecks in their recent ancestry. The value of wheat landraces in the production of stress‐tolerant varieties has been exploited by some researchers to study the mechanisms underlying boron (Paull et al., 1992), heat (Zhang et al., 2022) and drought tolerance (Lin et al., 2019; Naderi et al., 2020), but global landrace collections remain largely underutilized in the study of abiotic stress tolerance in wheat. Here, we present the YoGI landrace panel, and its accompanying transcriptome data, as a resource for researchers and breeders to utilize in the production of stress‐tolerant wheat varieties, and the study of stress tolerance mechanisms in this crucial crop.

In contrast with the extensively used methods of GWAS, RNA‐seq and microarray studies, network approaches, such as WGCNA, provide a more refined list of high‐impact candidate genes for further study. The role of these candidate genes as hubs within modules suggests they are likely to regulate the expression of a suite of genes, so manipulating their expression or activity will have far‐reaching effects on gene expression. The consequences of these global gene expression changes, as a result of hub gene manipulation, cumulate to produce a large phenotypic effect on the trait under study. Identifying, and manipulating, such hub genes is crucial if complex quantitative traits, such as abiotic stress tolerance, are to be altered in a significant way. These traits are influenced by hundreds or thousands of genes in the genome, so manipulating a poorly connected effector gene is likely to have little or no impact on the trait because of the action of other genes that remain undisturbed. These hub genes serve as valuable targets for breeders working on quantitative traits, as large‐scale changes in gene expression and, subsequently, phenotypic changes can be achieved via the targeted manipulation of a single hub.

Despite their seeming importance to breeders working on quantitative traits, such as thermotolerance, there is relatively little work on the use of network approaches to identify heat stress‐related hub genes in wheat (Girousse et al., 2018; Mishra et al., 2021). These works have facets in common that differ from the present work, however. First, the published works use RNA samples taken from plants exposed to stress as well as plants exposed to control conditions, whereas the present work uses only RNA from plants grown under control conditions for network construction. This means that although heat‐responsive hub genes will be missed in the present work, hubs whose expression under control conditions may predispose accessions to be more, or less, thermotolerant will be identified. A further difference is that the published works use far fewer different accessions (maximum of two) than the 337 landrace accessions used for network construction in the present work – a major difference in the volume of genetic diversity exploited.

The present work is also distinct from much of the above published works, as it concerns heat stress exposure and thermotolerance at an early developmental stage. The study of the genetic governance of wheat seedling thermotolerance is fairly limited, with only a handful of published works identifying key genes or genomic regions associated with the trait (Abd El‐Daim et al., 2014; Khan et al., 2022; Khatun et al., 2018). Much of the study on the effect of heat stress on wheat concerns its effect at, or around, anthesis and grain filling, and its subsequent effect on yield, as these are the developmental stages when heat stress commonly occurs in some major wheat producing countries (Stone & Nicolas, 1994, 1995, 1996). However, shifts in global temperature patterns has meant that months aligned with the early stages of spring habit wheat growth in many countries are becoming a lot warmer. For example, the March to May period (Northern Hemisphere’s meteorological spring) of 2022 was the fifth warmest on record (NOAA National Centers for Environmental Information, 2022a). In fact, May 2022 saw record temperatures reached across Southern, Central and Western Europe, whilst temperatures approaching record levels (in excess of 40°C in some cases) were observed in some Southern and North‐eastern states of the USA, a major producer of spring wheat (NOAA National Centers for Environmental Information, 2022a; NOAA National Centers for Environmental Information, 2022b). This shift in global temperature patterns has been accelerating in recent times with the ten warmest January to May periods all occurring since 2010 (NOAA National Centers for Environmental Information, 2022a), whilst, as predicted by climate variability studies (Easterling et al., 2000; Thornton et al., 2014; Haokip et al., 2020), periods of unseasonal spring heat stress are likely to occur more frequently in the coming years. Such periods have been observed in recent times – for example, May 2020 saw sustained daytime temperatures exceeding 30°C in the USA, Italy and Türkiye (NOAA National Centers for Environmental Information, 2020), whilst 2018 saw temperatures approaching record levels reached in major spring wheat producing states in the American Midwest, such as Minnesota which experienced temperatures of up to 38°C (NOAA National Centers for Environmental Information, 2018). The need, therefore, to prepare future wheat crops to tolerate heat stress during early development is imperative as spring temperatures continue to rise and unseasonal periods of extremely highly temperatures become more common around the world in the coming years.

Ensuring wheat varieties are thermotolerant during early development will not only protect crops against rising spring temperatures and unseasonable heat stress events, as recent evidence indicates accessions that are thermotolerant at the seedling stage also show higher yields when exposed to heat stress after anthesis (Lu et al., 2022). This work showed that there was a significant positive correlation between thermotolerance at the two developmental stages, and so suggests that breeding thermotolerant seedlings will also protect plants from heat stress yield damage if exposed to high temperatures later in development. These findings are particularly promising when viewed in tandem with the present work, as here we have presented three hub genes in which expression under control conditions can be used as markers of seedling thermotolerance, but perhaps also as markers of thermotolerance at yield.

The hub genes tested as predictive markers for early thermotolerance were selected based on their function, or on the function of their orthologous genes in other species, as well as their centrality within modules deemed to be particularly stress‐associated, thanks to their abundance of genes possessing stress‐associated GO terms. Three of the six hub genes were selected because of their roles as HSPs, and probable subsequent direct involvement in thermotolerance, whereas the remaining three hubs were selected because of their likely involvement in processes that may confer thermotolerance or improved growth under heat stress.

The three predictive hub genes were perhaps obvious actors in determining thermotolerance, because of their roles as HSPs. We hypothesized that *TraesCS4D01G207500.1* may act as a master regulator, and repressor, of thermotolerance in wheat because of its similarity to *AtHSP70‐1/Hsc70‐1*. Arabidopsis mutant lines showed increased basal thermotolerance, whereas the overexpression of *AtHSP70‐1/Hsc70‐*1 led to increased heat sensitivity, suggesting that the gene negatively influences thermotolerance (Tiwari et al., 2020). The protein acts on AtHsfA1d, AtHsfA13 and AtHsfA2, activators of thermotolerance, which subsequently represses the expression of the thermotolerance chaperone, *AtHSP101*. The hub gene was validated as a predictive marker of thermotolerance, but unlike its Arabidopsis orthologue, the gene appears to have a positive effect on thermotolerance, as accessions with higher levels of expression tended to be more thermotolerant (Figure 3).

*TraesCS7B01G149200.1* and its homeologue *TraesCS7D01G241100.1* have already been described as actors in the biotic‐stress response, and named *TaHSP90.2‐B1* and *TaHSP90.2‐D1*, respectively (Wang et al., 2011). However, we hypothesized that these genes may also play a positive role in thermotolerance, as the homeologues show sequence similarity to *AtHSP81.4*, a gene known to be highly expressed in the thermotolerant relative of Arabidopsis, *Thellungiella salsuginea* (Higashi et al., 2013; Taji et al., 2004). Moreover, the genes also share extreme sequence similarity to *TaHSP90*, a gene found to be highly expressed under heat stress in a heat‐ and drought‐tolerant wheat cultivar, C306, and which increased thermotolerance when overexpressed in *Escherichia coli*, suggesting that the gene plays a key role in determining thermotolerance in wheat (Vishwakarma et al., 2018). The hub genes have 99.9% and 97.4% similarity, respectively, with the *TaHSP90* sequence (accession no. MF383197) in this study, with *TraesCS7B01G149200.1* being the top BLAST hit against the IWGSC reference transcriptome. This allelic variation, likely a result of population structure differences between C306 and the Chinese Spring reference variety, does not affect amino acids within the active site of TaHSP90, and therefore is unlikely to affect protein function – suggesting that these hub genes fulfil the same function as *TaHSP90* and improve thermotolerance in wheat, as observed in the present work (Figure 3).

The three validated hub genes are all members of the same module, so their shared relationship between expression and early thermotolerance is likely to be a result of transcriptional co‐regulation. *TraesCS4D01G207500.1* was identified as the most central gene within the module, followed by *TraesCS7B01G149200.1* and *TraesCS7D01G241100.1* (Table S3), and we found that there was a significant relationship between *TraesCS4D01G207500.1* expression and the expression of the homeologous hubs, suggesting that *TraesCS4D01G207500.1* regulates the expression of the previously characterized thermotolerance hub genes.

We propose that *TraesCS4D01G207500.1* expression can be used as a marker of early thermotolerance because of its inferred function as a regulator of thermotolerance genes. As well as being connected to the characterized thermotolerance hub genes and validated markers, *TraesCS7B01G149200.1* and *TraesCS7D01G241100.1*, we found that *TraesCS4D01G207500.1* may also act to regulate the expression of a suite of other HSPs, being connected to 11 other HSPs as well as five members of the DnaJ HSP subfamily, with both groups of proteins heavily associated with the heat‐stress response and thermotolerance (Bourgine & Guihur, 2021). The hub was also connected to five heat‐shock transcription factors (HSFs), which suggests that, consistent with observations of its orthologue in Arabidopsis (Tiwari et al., 2020), the hub may be able to determine thermotolerance through the regulation of various HSFs. Here, however, we see that the increased expression of the hub is likely to lead to the upregulation of these genes and, subsequently, to increased thermotolerance. *TraesCS5A01G437900.1* is *TaHsfA2‐1*, a gene found to play a key functional role determining thermotolerance in wheat seedlings (Liu et al., 2020). *TraesCS5D01G445100.2* is *TaHsfA2e‐5D*, a transcription factor previously reported to confer heat and drought stress tolerance when expressed in yeast and Arabidopsis, via its activation of HSPs and other stress‐related genes (Bi et al., 2022). *TraesCS1A01G375600.2* is another HSF transcription factor (*TaHsfA6e*) that acts to regulate HSP expression as part of the thermotolerance network (Kumar et al., 2018). *TraesCS1B01G396000.3* and *TraesCS1D01G382900.1* are poorly characterized homeologues encoding *Triticum aestivum heat stress transcription factor A2c‐like*. As well as HSPs, HSFs and DnaJ proteins, the hub gene is also connected to all three homeologous genes that showed sequence similarity to *Triticum dicoccoides* serine/arginine‐rich splicing factor SR45a‐like, a pre‐mRNA splicing protein that plays a crucial role during the heat‐ and salt‐stress responses of plants (Li et al., 2021; Ling et al., 2021). The potential regulation of such genes by *TraesCS4D01G207500.1*, therefore, offers an explanation as to why its expression under control conditions is significantly associated with the degree of early thermotolerance in an accession.

In this work, we identified three stress‐associated hub genes hypothesized to govern thermotolerance, before confirming a significant association between hub gene RPKM and normalized loss in mean dry biomass in several landrace accessions. Two of these validated hub genes are almost identical to a thermotolerance gene already characterized in wheat; however, upon further investigation of the hubs, we found that *TraesCS4D01G207500.1* is likely to act upstream of these genes, regulating their expression, and explaining the similarity in the relationship between hub gene RPKM and early thermotolerance shown for each of these three hubs. Unlike its Arabidopsis orthologue (*AtHSP70‐1/Hsc70‐1*), however, *TraesCS4D01G207500.1* appears to positively influence thermotolerance. As well as potentially regulating the expression of the other hubs, it appears that *TraesCS4D01G207500.1* confers increased early thermotolerance via the probable regulation of the expression of a suite of other HSPs, a stress‐responsive splicing factor and HSFs previously shown to improve thermotolerance in wheat. In addition to this, we were also able to identify six other stress‐associated master regulator hub genes that may serve as good targets for thermotolerance improvement, but could not be validated in the present work. If the findings of recent work (Lu et al., 2022) apply here, the expression of these validated hub genes may not only serve as markers for early thermotolerance, but also as markers for tolerance to heat stress at anthesis – potentially allowing breeders to make predictions about heat stress‐related yield losses from a very early developmental stage. The present work, therefore, presents an exciting step forward towards the production of thermotolerant wheat varieties able to tolerate periods of high temperatures both early and late in development, and provides breeders new validated targets to aid them in this goal.

Here we have introduced the YoGI landrace panel as an important resource for wheat breeders, due to it's extensive genetic and phenotypic diversity. We have shown how the landrace panel can be used successfully to aid the production of more stress‐tolerant wheat varieties, as in the present work the expression levels of three stress‐related hub genes were shown to be significantly associated with the thermotolerance of landrace accessions during vegetative development. The present work not only validates these genes as predictive markers for use in breeding programmes, but also suggests that expression of *TraesCS4D01G207500.1* is able to determine the degree of thermotolerance of an accession through potential action as a master regulator over the expression of an array of HSPs and HSFs, including the other validated hub genes, which show remarkable similarity to a previously characterized thermotolerance wheat gene.

## EXPERIMENTAL PROCEDURES

### YoGI landrace panel

The YoGI panel constitutes 342 *T. aestivum* accessions sourced from wheat collections held at the Germplasm Resource Unit (GRU, https://www.jic.ac.uk/research-impact/germplasm-resource-unit), the International Maize and Wheat Improvement Center (Centro Internacional de Mejoramiento de Maíz y Trigo, CIMMYT, https://www.cimmyt.org) and the Crop Research Institute (Výzkumný Ústav Rostlinné Výroby, https://www.vurv.cz) (Table S5). Landraces were selected to maximize the diversity and representation of countries across the global wheat mega‐environments (Sonder, 2016), including both spring and winter habit accessions. A single plant from each accession was grown for RNA extraction, with the second or third leaf harvested from seedlings at the midpoint of the day. RNA was extracted using the E.Z.N.A Plant RNA kit (Omega Bio‐Tek, https://www.omegabiotek.com), according to the manufacturer's instructions, including the recommended DNase I step. Leaf transcriptome data were generated at the Oxford Genomics Centre (https://www.ogc.ox.ac.uk) using the Illumina HiSeq platform (https://www.illumina.com). Reads were trimmed to a length of 127 bp with fastx_trimmer (Gordon & Hannon, 2010) to meet the requirements of maq. The trimmed reads were mapped by maq (Li et al., 2008) to the IWGSC RefSeq transcriptome v1.0 representative CDS models, with default parameters, meaning that reads with no more than two mismatches with summed *Q* ≥ 70 were mapped. Using the perl scripts described by Higgins et al. (2012), transcript abundance was quantified and normalized as RPKM value for each CDS model for each sample. All plants used in subsequent experiments were grown from selfed seed from the sequenced plant.

### Transcriptome display tile plots

The RPKM values for each of the homoeologous triplets were rescaled between 1 and 0, where the individual with the lowest RPKM value = 1 and the individual with the highest expression value = 0. These values were then converted to RGB hexcodes, where the A genome homoeologue is represented in cyan, the B genome in magenta and the D genome in yellow, as previously described (Harper et al., 2016; He et al., 2017). In addition to the landraces, we included representative AA (*Triticum urartu*), BB (*Aegilops speltoides*) and DD (*Aegilops tauschii*) genome diploid species samples, and produced tetraploid (AABB, BBDD, AADD) and hexaploid (AABBDD) lanes *in silico* to aid with the analysis of colour variations.

### Co‐expression network construction and module detection

The wgcna package (Langfelder & Horvath, 2008, 2012) was employed to construct a co‐expression network in r 3.6.3 (R Core Team, 2021) using RPKM data from the YoGI landrace panel. Five accessions were removed after sample clustering, whereas 16 733 genes were removed for too many zero values, leaving 94 057 genes from 337 accessions for network construction. The ‘blockwiseModules()’ function conducted blockwise network construction according to the default parameters of the function, except the following: maximum block size = 5000; soft threshold power = 8 (the first power to exceed a scale‐free topology fit index of 0.85); minimum module size = 30; and merge cut height = 0.25. After module detection, edge and node files were created using the ‘exportNetworkToCytoscape()’ function with a threshold of 0.1, filtering out weak connections between genes (nodes).

### GO term enrichment analysis

To identify the GO terms significantly enriched in each module, the genes present in each module were collated and submitted to the agriGO Singular Enrichment Analysis tool (Du et al., 2010; Tian et al., 2017). A Fisher's exact test was performed for each module, as GO terms possessed by the genes in each module were compared against a background of GO terms possessed by all of the genes used for network construction, with *P* =0.05 as the threshold, Hochberg (FDR) as the multi‐test adjustment method (Benjamini & Hochberg, 1995) and with a minimum number of five mapping entries. A GO term was considered enriched in a module when its FDR‐adjusted *P*‐value was <0.05. GO annotation terms of IWGSC RefSeq transcriptome v1.0 were retrieved from: https://opendata.earlham.ac.uk/wheat/under_license/toronto/Ramirez‐Gonzalez_etal_2018‐06025‐Transcriptome‐Landscape/data/TablesForExploration/FunctionalAnnotation.rds (Ramírez‐González et al., 2018).

### Network visualization and hub identification

To calculate the degree (connection) scores for each gene, network modules were either visualized in cytoscap 3.9.1 (Shannon et al., 2003) and analysed using the cytoscape network analyser tool (Assenov et al., 2008), or the number of connections to and from each gene were counted in r. Genes in a module that showed the highest degree scores (most connections) were deemed to be the central hubs (Table S3). In some cases, however, multiple genes in one module shared the highest degree score, whereas in other modules, the most well‐connected gene was poorly characterized, with little known about its function. In these cases, the characterization of genes was used to determine which of the other well‐connected genes in each module would most likely have a regulatory role on gene expression or physiological processes, based on our knowledge of the function of the gene, or our knowledge of an orthologous gene in other species, such as Arabidopsis. In these instances, genes that had high degree scores and appeared to potentially play a regulatory role were selected as hub genes for further study. In other modules, multiple promising candidates were amongst the most well‐connected genes, with only small differences in degree score between them. In this case, all well‐connected promising candidate genes were taken forward for further study.

### Hub gene validation

After hub gene identification, the expression of these genes were tested as markers for early thermotolerance in spring habit accessions. All hub genes with a mean value of RPKM > 1 across the entire panel were taken forward as potential markers (*n* = 6). Winter habit accessions were not used in validation experiments to prevent potential complications regarding heat‐stress exposure during early development interfering with the need to vernalize winter habit accessions.

A total of 15 spring habit landrace accessions were selected from the panel for use in the thermotolerance plant growth assay, based on their expression of the six hub genes (Table S4). We hypothesized that because these accessions showed a range of hub gene RPKM values, they would also show varying degrees of early thermotolerance and that there would be a significant relationship between the two factors. If such a relationship was observed, it would suggest that expression of the hub gene could be used as a marker of early thermotolerance.

Seeds of these accessions were sown in Levington Advance Seed & Modular F2S compost (https://www.lovethegarden.com) mixed with Aggregate Industries Garside Sands 16/30 sand (80:20 ratio; https://www.aggregate.com) treated with CaLypso insecticide (0.083 ml mixed with 100 ml water, applied to each litre of compost; https://cropscience.bayer.com). The seeds used in the present work were from plants selfed through at least three generations. Plants were grown in an AR‐75L2 growth cabinet (Percival, https://www.percival‐scientific.com/products/) at 22°C/16°C (day/night) on an 18 hour day/night cycle. All plants were exposed to these control conditions until the three‐leaf stage of development was reached. At this point, half the replicates of each accession were moved to a separate Percival AR‐75L2 growth cabinet and exposed to 35°C/30°C (day/night), with all other conditions being the same as in the control cabinet. Stressed plants were exposed to these conditions for 14 days following the three‐leaf stage, before being returned to control conditions for 3 days to serve as a recovery period. The remaining half of the replicates of each accession remained under control conditions for the duration of the experiment. All plants were harvested 17 days after they had reached the three‐leaf stage, by cutting the stem at the soil surface. Dry biomass measurements were taken after harvested tissue was dried at 65°C for 2 days. With the size of the cabinets, a block design was employed whereby two blocks each contained four replicates of each accession in each condition, with the same cabinets being used for both blocks of the experiment.

### Data processing and analysis

After all the data had been collected, outliers were identified and removed from the data set using Tukey's method, whereby all values more than 1.5 interquartile range (IQR) away from the first and third quartile are removed (Tukey, 1977). After data processing had removed the extreme outliers from the data set, the remaining dry biomass measurements were used to calculate mean trait scores for each accession in each condition, from which normalized biomass loss scores, between stressed and control conditions, were calculated as follows: 1 – (mean control dry biomass/mean stressed dry biomass). The normalized loss of mean dry biomass was used as a measure of thermotolerance, with tolerant accessions showing less biomass loss between conditions, and vice versa. To determine whether there was a significant relationship between the expression of any of the hub genes and dry biomass loss, a linear regression analysis was performed. Similarly, to test whether there was a significant relationship between the expression of hub genes within the same module, linear regression analysis was again employed. A relationship was considered significant when *P* < 0.05.

## AUTHOR CONTRIBUTIONS

LJB, ALH, IB and SMM^†^ conceived and planned the project. LJB, ALH, AF and LC performed plant growth experiments, and LJB and ZH conducted transcriptomic analyses. LJB, ALH and ZH wrote the article, and all authors reviewed it. ^†^SMM died on 5 June 2022.

## CONFLICT OF INTEREST

The authors declare that they have no conflicts of interest associated with this work.

## Supporting information


**Figure S1.** Expression of remaining hub genes were not significantly associated with early thermotolerance.

**Table S1.** Mapping statistics for YoGI panel.

**Table S2.** Normalized transcript abundance (RPKM) for the YoGI panel.

**Table S3.** Stress‐associated hub gene module membership, degree score and module size.

**Table S4.** Normalized mean dry weight loss and RPKM values for each of the six hub genes for each accession used in the validation experiment.

**Table S5.** YoGI panel accession information.

## Data Availability

Raw sequence read data from this article can be found in the SRA data library under accession number PRJNA912645. Normalized transcript abundance (RPKM) for each of the landraces and diploid relatives is provided in Table S2.
