# Supplementary figures and images for: Co‐expression network analysis of diverse wheat landraces reveals markers of early thermotolerance and a candidate master regulator of thermotolerance genes
Source: Plant J. 2023 May 20;115(3):614–26. doi: 10.1111/tpj.16248 (PMC10953029; doi:10.1111/tpj.16248)

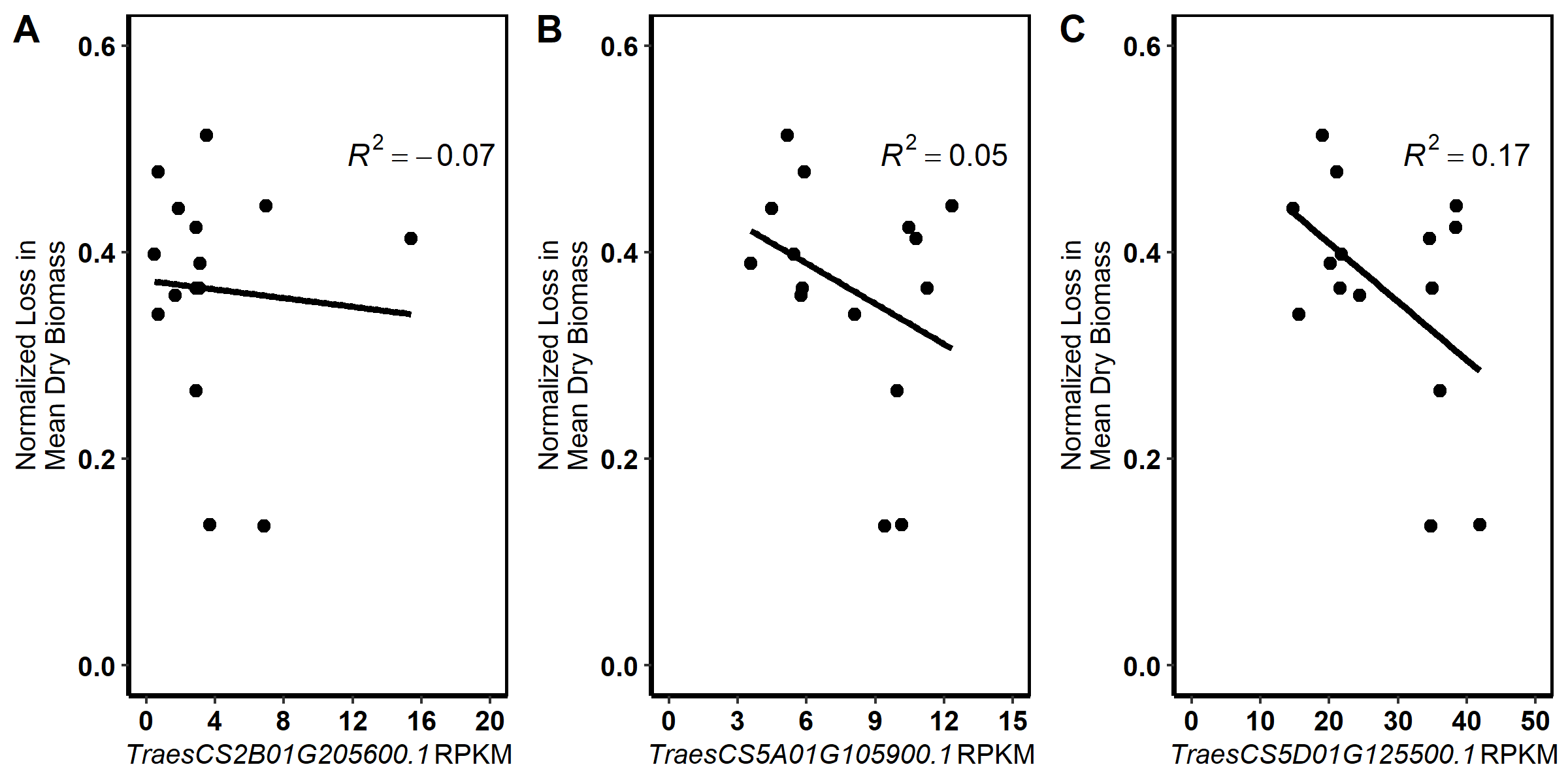

Supplement: Supplementary file 1 — Figure S1. Expression of remaining hub genes were not significantly associated with early thermotolerance. [file TPJ-115-614-s006.png]
